# Prefrontal Cortex and Amygdala Subregion Morphology Are Associated With Obesity and Dietary Self-control in Children and Adolescents

**DOI:** 10.3389/fnhum.2020.563415

**Published:** 2020-12-03

**Authors:** Mimi S. Kim, Shan Luo, Anisa Azad, Claire E. Campbell, Kimberly Felix, Ryan P. Cabeen, Britni R. Belcher, Robert Kim, Monica Serrano-Gonzalez, Megan M. Herting

**Affiliations:** ^1^Center for Endocrinology, Diabetes and Metabolism, Children’s Hospital Los Angeles, Los Angeles, CA, United States; ^2^Department of Pediatrics, Keck School of Medicine, University of Southern California, Los Angeles, CA, United States; ^3^The Saban Research Institute at Children’s Hospital Los Angeles, Los Angeles, CA, United States; ^4^Department of Medicine, Keck School of Medicine, University of Southern California, Los Angeles, CA, United States; ^5^Department of Psychology, University of Southern California, Los Angeles, CA, United States; ^6^Department of Preventive Medicine, Keck School of Medicine, University of Southern California, Los Angeles, CA, United States; ^7^Laboratory of Neuro Imaging, USC Mark and Mary Stevens Neuroimaging and Informatics Institute, Keck School of Medicine, University of Southern California, Los Angeles, CA, United States; ^8^Department of Pediatrics, Warren Alpert Medical School of Brown University, Providence, RI, United States

**Keywords:** obesity, adolescence, dietary control, prefrontal cortex, amygdala

## Abstract

A prefrontal control system that is less mature than the limbic reward system in adolescence is thought to impede self-regulatory abilities, which could contribute to poor dietary choices and obesity. We, therefore, aimed to examine whether structural morphology of the prefrontal cortex (PFC; involved in cognitive control) and the amygdala (a key brain region for reward-related processing) are associated with dietary decisions and obesity in children and adolescents. Seventy-one individuals between the ages of 8–22 years (17.35 ± 4.76 years, 51% female, 56% were overweight or obese) participated in this study; each participant completed a computer-based food choice task and a T1- and T2-weighted structural brain scans. Two indices of obesity were assessed, including age- and sex-specific body mass index (BMIz) and waist-to-height ratio (WHtR). The behavioral task included rating 60 food stimuli for tastiness, healthiness, and liking. Based on each participant’s self-ratings, 100 binary food choices were then made utilizing a computer mouse. Dietary “self-control” was calculated as the proportion of trials where the individual chose the healthier food item (vs. the tastier food item) over the total number of trials. Cortical thickness and amygdala subnuclei volumes were quantified using FreeSurfer 6.0 and CIT168 atlas, respectively. We found that WHtR was negatively associated with the thickness of bilateral superior frontal, left superior temporal, right insula, and right inferior temporal regions (*p* < 0.05, corrected for multiple comparisons). We also found WHtR to be positively associated with the volume of the central nucleus (CEN) region of the amygdala (*p* = 0.006), after adjusting for the hemisphere, age, sex, and intracranial volumes. A similar data pattern was observed when BMIz was used. Moreover, we found that across all participants, thinner right superior frontal cortex and larger left CEN volumes predicted lower dietary self-control. These results suggest that differential development of the PFC and amygdala relate to obesity and dietary self-control. Further longitudinal studies are merited to determine causal relationships among altered PFC to amygdala neural circuitry, dietary self-control, and obesity.

## Introduction

Obesity is a common and serious public health problem, with a prevalence of 18.5% in youth between 2 and 19 years old in the United States (Ogden et al., 2010). Obesity affects 13.7 million children and likely persists into adulthood as a risk factor for cardiovascular disease and type 2 diabetes (Pi-Sunyer, 1991; Singh et al., 2008; Biro and Wien, 2010). Even though obesity occurs in a state of chronic positive energy balance (i.e., energy intake greater than energy expenditure), the origins are multifactorial and include environmental and genetic factors that could impact the central regulation of food intake and energy homeostasis (Timper and Brüning, 2017). Many genes associated with obesity are preferentially expressed in the central nervous system (Locke et al., 2015), suggesting that the brain plays an important role in the regulation of energy intake and expenditure. Functional neuroimaging studies in children and adolescents indicate that obesity may involve dysregulation of two key neural systems: (1) hypo-functioning of the prefrontal cortex (PFC) involved in inhibitory control of appetitive food rewards (Batterink et al., 2010; Bruce et al., 2013; Carnell et al., 2017; Jensen et al., 2017; Luo et al., 2019); and (2) hyper-reactivity of the limbic system involved in reward and emotion processing of external food cues (Boutelle et al., 2015; Rapuano et al., 2016).

The PFC plays an important role in cognitive control, including top-down regulation of appetite. A recent meta-analysis on functional neuroimaging studies involving tasks that probe different aspects of dietary self-control reported that the inferior frontal gyrus (IFG) and middle frontal gyrus (MFG) were among the regions that showed reduced activation during self-control as a function of body mass index (BMI) in healthy adults (Han et al., 2018). Similar negative relationships have been observed in studies of healthy children and adolescents (Batterink et al., 2010; Bruce et al., 2013; Carnell et al., 2017; Jensen et al., 2017; Luo et al., 2019). There have been inconsistent findings regarding relationships between PFC cortical thickness and BMI in children, although recent large-scale studies have shown an *inverse* correlation between BMI and cortical thickness, with the greatest correlation observed in the PFC (Laurent et al., 2020; Ronan et al., 2020). Waist circumference [i.e., waist-to-height ratio (WHtR), or waist-to-hip ratio] has also been studied concerning the brain (Ross et al., 2015; Hamer and Batty, 2019; Ronan et al., 2020), as an index of central obesity that is associated with cardiovascular risk factors (Katzmarzyk et al., 2012). A recent study in children reported additional brain clusters were observed when WHtR was used instead of BMI, suggesting central obesity markers may have better sensitivity in detecting obesity-linked morphology (Ronan et al., 2020). Thus, findings from previous studies indicate that more research is needed on the relationships between PFC activity and different markers of body composition in youth, whose “top-down” processes are rapidly developing.

The limbic system includes regions that are important in the central regulation of feeding behavior in humans. The amygdala is located in the anterior temporal lobe, with early studies showing that bilateral damage to the temporal lobe leads to hyperphagia and obesity in human and animal models (Sawa et al., 1954; Weiskrantz, 1956; Green et al., 1957; Koikegami et al., 1958; Wood, 1958; Wilkinson and Peele, 1962; Kling and Dunne, 1976). Thus, it was suggested that the amygdala may be essential in regulating feeding behavior (Berthoud, 2012). Functional magnetic resonance imaging (fMRI) studies have shown that children with obesity exhibit hyper-responsivity to food rewards in the amygdala compared to lean children (Boutelle et al., 2015), and the basolateral amygdala response to food cues in young adults is associated with future weight gain (Sun et al., 2015). Structural studies have primarily focused on the whole amygdala, but further study is merited of its heterogeneous nuclei in the amygdala that have distinct cytoarchitecture, connectivity, and function that are involved in a range of behaviors including emotion, arousal, and stimulus reward learning. Four specific nuclei implicated in feeding behavior have been identified, including the lateral nucleus (LA), dorsal and intermediate basolateral (BLDI), central nucleus (CEN), and cortical and medial nuclei (CMN). The LA is anatomically connected to the lateral hypothalamus, receiving input *via* a pathway critical for “cue-initiated” feeding (Petrovich and Gallagher, 2003). Notably, the basolateral complex of the amygdala (which includes both the BLDI and LA) is important for reward learning (e.g., linking objects such as food, with reward value; Wassum and Izquierdo, 2015). The hypothalamus, also a part of the limbic system, is one of the most important regions involved in the central regulation of metabolism, and these interactions between the hypothalamus and amygdala help regulate food intake. As well, there is emerging overlap of metabolic and emotional pathways, with the nuclei of the hypothalamus and amygdala activated by hunger and fear (Comeras et al., 2019). Lesions of the CEN lead to the disruption of inhibitory control of eating in the presence of an aversive cue (Petrovich et al., 2009; Prévost et al., 2012) and stress-induced obesity may induce insulin resistance in the central amygdala, involving neuropeptide Y neurons (Ip et al., 2019). There are also bidirectional projections between the medial amygdala and medial hypothalamus *via* the stria terminalis, and disruption of this pathway has been shown to lead to hyperphagia and obesity (King et al., 2003). These findings suggest that specific amygdala nuclei may be important in regulating feeding behavior. Yet, their potential roles in food decision-making and obesity in humans remain largely unexplored.

Therefore, alterations in these PFC and amygdala circuits may provide a neurobiological link to obesity and/or impaired dietary self-control. Yet, it is unknown whether these impairments may begin to present themselves during child and adolescent development. Patterns of neurodevelopment have helped to create a newly evolving definition of adolescence from 10 to 24 years of age (Sawyer et al., 2018), a period that includes rapid maturation of the PFC and amygdala, yet with a protracted development of the PFC relative to limbic regions (Casey et al., 2008). Thus, the relative timing of neurodevelopmental processes may especially render adolescents vulnerable to poor dietary choices with potentially lifelong effects. Adolescents commonly make poor dietary decisions, such as consuming more fast food and refined sugars than any other age group (Nielsen et al., 2002; Bremer and Lustig, 2012). As such, the differential development of PFC and limbic circuitry could make adolescents particularly susceptible to sub-optimal dietary decision-making, and thereby contribute to the development or sustainment of obesity.

The goal of the current study was to examine whether brain morphology of the PFC, as characterized by cortical thickness, and volume of the amygdala related to obesity and dietary choice in a typical cross-sectional sample of youth with ages spanning late childhood to early adulthood to assess the entire developmental period of adolescence. We hypothesized that larger amygdala and thinner PFC would be linked to both obesity and poorer dietary self-control.

## Materials and Methods

### Study Participants

We studied 71 children and adolescents between the ages of 8–22 years (17.35 ± 4.76 years, 51% female, 56% were overweight or obese; Table 1). Participants were recruited at Children’s Hospital Los Angeles (CHLA), through previous participation in another University of Southern California (USC) research study, *via* flyers posted around the greater Los Angeles metro area, as well as, at community outreach events. Inclusion criteria included: being 8–22 years old, English as their primary language, and being otherwise healthy. Exclusion criteria included: a history of traumatic brain injury; a history of a neurological disorder; current or persistent psychiatric condition; a history of and persistent severe learning disorder, mental retardation, pervasive developmental disorder, or other condition requiring repeat or persistent specialized education; non-correctable vision or hearing, or sensorimotor impairments; and/or MRI contraindications. Participants 18 or older were also excluded if they reported a high level of lifetime exposure to drugs and alcohol based on NIAAA criteria (National Institute on Alcohol Abuse and Alcoholism (NIAAA)., 2011). This study was approved by the IRB Human Subjects Protection Office of USC and CHLA (CHLA-14-00191 and HS-16-00978). Parents and participants gave written informed consent and age-appropriate assent following the World Medical Association Declaration of Helsinki.

**Table 1:** Study population characteristics.

Characteristic	Overall (*N* = 71)
Age, Mean (SD)	17.35 ± 4.76
Male, %	49
Right Handedness, %	89
BMI Z-Score, Mean (SD)	0.89 ± 0.88
BMI Percentile, Mean (SD)	75.02 ± 23.98
Waist-to-height ratio, Mean (SD)	0.51 ± 0.07
Maternal Education, %
≤ High School	27
College/Associates	23
Bachelor	30
Master/Doctorate	19
Not Reported	1
Race, %
Caucasian	49
African American	7
Other	44
Ethnicity, %
Hispanic or Latino	46

### Anthropometric Measurements

Participant height (cm) and weight (kg) were measured using a stadiometer and calibrated digital scale respectively. BMI was calculated (kg/m^2^), and BMI percentile and BMI *z*-score for youth were determined based on the U.S. Center for Disease Control normative data (Kuczmarski, 2002). Waist circumference was measured at the midpoint between the iliac crest and lower costal margin in the midaxillary line, and WHtR was calculated.

### Neuroimaging

#### Magnetic Resonance Imaging Acquisition

All whole-brain T1- and T2-weighted MRI scans were collected on a Siemens Magnetom Prisma 3 Tesla MRI scanner using a 32-channel head coil at the University of Southern California’s Center for Image Acquisition. The 3D T1 and T2 weighted structural images were acquired using sagittal whole-brain MPRAGE sequences (T1: TR = 2,400 ms, TE = 2.22 ms, flip angle = 8°, BW = 220 Hz/Px, FoV = 256 mm, 208 slices, and 0.8 mm isotropic voxels, with a GRAPPA phase-encoding acceleration factor of 2; T2: TR = 3,200 ms, TE = 563 ms, BW = 744 Hz/Px, FoV = 256 mm, 208 slices, 0.8 mm isotropic voxels, and 3.52 ms echo spacing, with a GRAPPA phase-encoding acceleration factor of 2). The T1 sequence lasted 6 min and 38 s, and the T2 sequence lasted 5 min and 57 s. All scans were reviewed by a radiologist for incidental findings of abnormalities and all images underwent visual quality control to assess for motion and were rated on a 3-point scale of Pass, Check, Fail (Backhausen et al., 2016). All 71 included participants were rated as Pass or Check before preprocessing; four participants that were originally collected for this study, did not have usable data and were excluded from this analysis (i.e., Fail and were not included).

#### Whole Brain Segmentation

Cortical parcellation was performed using each subject’s T1- and T2-weighted images using FreeSurfer^1^ v6.0 (Fischl et al., 2002; Reuter et al., 2012). The surface-based pipeline was used to estimate cortical thickness, as follows (Dale et al., 1999; Fischl and Dale, 2000; Fischl et al., 2002). Each subject’s T1- and T2-weighted images were registered to MNI305 and skull-stripped. White matter was segmented by classifying voxels as white matter or other based on the intensity at each voxel. For each hemisphere, a white surface (the boundary between white and gray matter) and pial surface (the boundary between gray matter and CSF) was defined, and the distance between the two computed whole-brain cortical thickness. After segmentation, all images were inspected by a trained operator to assess the accuracy of segmentation for each participant (AA). No manual intervention was performed. In addition to the surface-based stream, the intracranial volume (ICV) from the volume-based pipeline was extracted as a covariate in models examining amygdala volumes.

#### Amygdala Segmentation

The amygdala was segmented into subregions using the CIT168 atlas (Tyszka and Pauli, 2016); previously published manuscripts describe the creation, validation, and estimation of individual differences, and also compare the CIT168 with previous atlas (Tyszka and Pauli, 2016; Pauli et al., 2018). In the creation and validation of the CIT168 atlas, Tyszka and Pauli (2016) established that a Contrast to Noise (CNR) ≥1 is necessary for robust volume estimation of the ground truth volume. Thus, the intensity contrast within each hemisphere of the amygdala was estimated from the interquartile range (IQR) of intensities within the entire amygdala from each subject’s T1-weighted image. The standard deviation (SD) of the noise was estimated from the residual signal obtained from the subtracted T1-weighted atlas template image from each subject’s T1-weighted image. The IQR was then divided by the mean residual noise SD to generate the CNR. For our amygdala data, the average amygdala CNR was 1.12 in our T1w and 1.04 in T2w data. Amygdala subregions were estimated at the individual level for each participant as previously published (Herting et al., 2020). Each participant’s T1w and T2w images were first registered to one another, then a single B-spline bivariate symmetric normalization (SyN) diffeomorphic registration algorithm from ANTs (Avants et al., 2007) was used to map to the CIT168 atlas using a joint cross-correlation similarity metric with equal weighting of the T1w and T2w image of the subject. We then used the inverse diffeomorphism to produce a probabilistic segmentation of each participant’s left and right entire amygdala, as well as each of the nine bilateral subfield regions of interest (ROI). Based on the existing literature, we focused our analysis on four *a priori* ROIs that have been implicated in appetite regulation, including the LA, BLDI, CEN, and CMN. The volume of the total amygdala and each subnuclei ROI for both hemispheres were extracted for analyses.

### Behavioral Food Choice Task

All participants completed a behavioral food choice task in a fasted state (at least 4 h, but the majority fasted overnight). The task was modified for youth from a food choice task previously employed in adults (Sullivan et al., 2015). The computer task was coded in MATLAB (version 2014a, Natick, MA, USA), and displayed with Psychophysics Toolbox^2^ (version 3). Instructions were designed to be at a 3rd-grade reading level, with visual cartoons and shorter phrases, and were read aloud to participants who were younger than 18 years old to standardize for literacy. During the task, participants were instructed to raise their hands with questions.

Thirty high-calorie and 30 low-calorie food cues were selected from two databases (Page et al., 2011; Blechert et al., 2014) and matched overall between calorie groups for red/green/blue color proportion, size, brightness, contrast, complexity, and normalized complexity. The high- and low-calorie food stimuli only differed by their caloric density (kcal/100 g). Food cues were selected to be foods familiar and appealing to a pilot group of youth.

#### Food Rating Task

Participants rated each of the 60 food cues for tastiness, healthiness, and overall desire (liking) to eat the food. Block and stimulus orders were randomized across participants. Ratings were made on the computer keyboard, using a 5-point scale with emoticons and words. Response time (*RT*) in this task is defined as the time between the initial presentation of a food cue and the subject’s keyboard response.

#### Food Choices Task

One hundred binary choices were presented based on the participant’s individual ratings for taste and health from the Food Ratings Task. Among the one hundred choice pairs, 75 were “challenge trials,” where one food had a higher tastiness rating yet lower healthfulness rating than the other food item (which thus had a higher healthfulness rating and lower tastiness rating). These challenge trials were deemed to present a self-control challenge to the participant.

Participants were counseled to keep the health of foods in mind, with the instructions for the task including a reminder slide to “try to keep it healthy,” to increase the frequency at which they exhibited dietary self-control in the task (Hare et al., 2011; Sullivan et al., 2015). They were also informed that one of their choices would be rewarded to them at the end of the task, incentivizing careful decision-making. In each trial of the task, participants chose the item they would like to eat using the cursor. Before starting the task, participants completed several practice trials.

Each trial was presented as follows: (1) a display box labeled “START” appeared at the bottom center of a black screen; (2) participants were required to click this box to begin the trial; (3) a blank screen of variable duration (200–500 ms; mean = 350 ms) then appeared; (4) the cursor would reappear in the bottom center of the screen and two food pictures were pseudo-randomized to appear with one at the left and one at the right upper corner; and (5) participants selected the food they preferred by moving the cursor continuously to the box that contained their preferred food. A participant’s RT was recorded as the time between food cue presentation and choice. Fluid cursor movements were promoted by the food cues not being displayed until the mouse movement was detected. To further encourage smooth and direct mouse movements, if the participant took longer than 4 s to make a choice, a message prompting faster decisions was displayed. Trials were separated by a fixation cross of random duration (400–700 ms; mean = 550 ms). During choice, the *x, y* coordinates of the mouse cursor were recorded at a temporal resolution equal to the screen refresh rate (67 Hz).

At the end of the task, a computer algorithm selected a food item (out of six different items in stock) that corresponded to one of the food items selected by the participant during the Food Choices task. The chosen food item was displayed on the screen and then offered to the participant at the end of the computer task.

Dietary self-control was considered successful when the healthier food cue in the challenge trial was chosen. An individual measure of overall dietary self-control, the “self-control success ratio (SCSR)” was computed as the proportion of challenge trials per participant in which dietary self-control was successful.

### Statistical Analysis

Whole-brain vertex-wise cortical analyses were performed using Freesurfer’s Query, Design, Estimate, Contrast (Qdec) interface including the main predictor of WHtR while also accounting for age and sex, and results were corrected for multiple comparisons by the method of Monte Carlo simulation with *p* < 0.05 (Hagler et al., 2006). For each identified significant cluster, the mean thickness was extracted for each participant to perform follow-up analyses in association with behavior. All behavioral statistical evaluations of the data were performed in R (version 3.6; r-project.org, Vienna, Austria). Linear regression, as well as the linear mixed-effects model (LME) analyses, were conducted using the R package “nlme.”

In all analyses, a model reduction strategy was applied to first examine a potential WHtR by age interaction. However, in all analyses, the interaction term was found not to be significant. Thus, the interaction was removed, and the final model included the main effects of WHtR while adjusting for age.

For the behavioral task, LME analyses included examining how WHtR related to ratings of the food stimuli and RT during food choices with the repeated measures including food attributes (tastiness, healthiness) and calorie content of the food (high-calorie, low-calorie), with subject as a random factor. A separate model was performed to examine how WHtR and age related to liking ratings.

For amygdala volumes, separate LME analyses examined how WHtR related to volumes of the total amygdala or *a priori* subregions of interest (LA, BLDI, CMN, CEN), while including age, sex, hemisphere (left, right), and ICV as covariates, and subject as a random factor. *Post hoc* tests were completed using the package “reghelper” to calculate the simple slopes of interaction at the mean level of any continuous covariates.

Following *a priori* analyses, we aimed to also examine the link between obesity-related brain correlates with behavior, we examined if identified cortical and amygdala correlates and their potential interactions predicted SCSR on the food task while adjusting for WHtR, age, sex, and ICV. Last, we implemented a stepwise regression using both forward and backward selection to examine the best brain predictors in a single model to determine the best predictors of the SCSR on the food choice task. For completeness, we also explored models using BMIz in place of WHtR (Supplementary Figure 2, Supplementary Tables 3–5).

## Results

### Relationships Between WHtR, Cortical Thickness, and Amygdala Volumes

**Figure 1:**
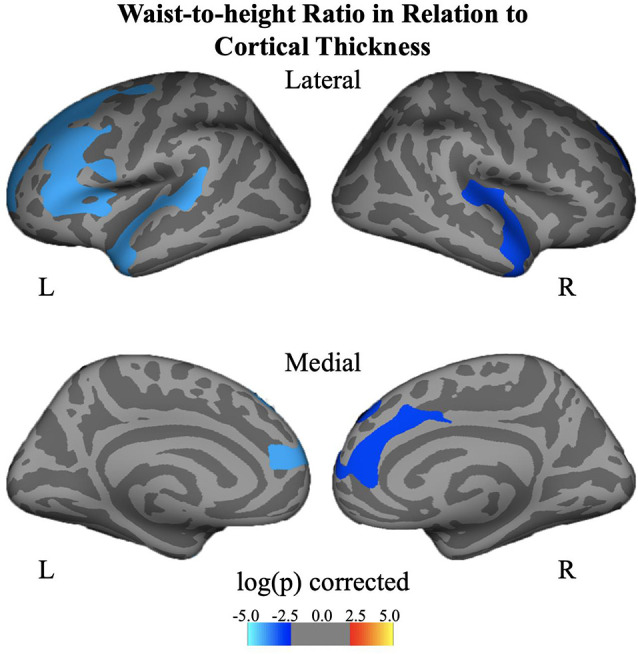
Associations between waist-to-height ratio and cortical thickness, controlling for age and sex and corrected for multiple comparisons at *p* < 0.05.

**Figure 2:**
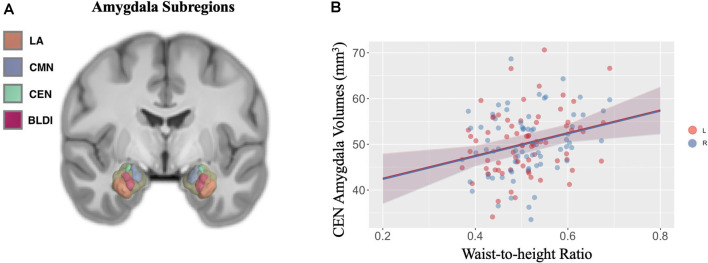
**(A)** Visual display of four *a priori* subregions of amygdala: lateral nucleus (LA) in orange, cortical, medial nuclei (CMN) in blue, central nucleus (CEN) in green, and dorsal and intermediate basolateral (BLDI) in dark pink. **(B)** Waist-to-height ratio is associated with larger left (denoted by pink solid circles) and right (denoted by blue solid circles) CEN amygdala volumes.

Negative associations were seen between cortical thickness and WHtR, controlling for age and sex, including significant clusters in the bilateral superior frontal, left superior temporal, right insula, and right inferior temporal regions (Figure 1, Table 2). There was a trend association between WHtR and overall amygdala volume after adjusting for the hemisphere, age, sex, and ICV (Table 3). In subregion analyses, we found a main effect of WHtR on the volume of the CEN (Figure 2), but not the CMN, BLDI, or LA of the amygdala, after adjusting for the hemisphere, age, sex, and ICV (Table 3).

**Table 2:** Main effect of waist-to-height ratio on cortical thickness (*N* = 71).

Cluster location	Hemisphere	Cluster size (mm^2^)	MNI coordinates			T Max
			*X*	*Y*	*Z*	
Superior Frontal Cortex	L	8,260	−20.1	25.2	39.4	−3.794
Superior Temporal Cortex	L	2,691.45	−49.7	8	−14.8	−3.398
Insula Cortex	R	2,422.01	35.2	−24.8	5.4	−3.802
Superior Frontal Cortex	R	2,306.41	14	36.5	11.3	−3.394
Inferior Temporal Cortex	R	1,785.23	43.6	−23.9	−17	−3.302

*Controlled for age and sex and corrected for multiple comparisons (*p* < 0.05). Abbreviations: L, left; R, right*.

**Table 3:** Associations between waist-to-height ratio and amygdala volumes (*N* = 71).

Volumes (mm^3^)	Beta estimates	95% CI	*p*
Total Amygdala	483.87	−65.07 to 1,032.82	0.083
BLDI	54.58	−12.41 to 121.57	0.11
CEN	24.88	7.42 to 42.33	**0.006**
CMN	29.39	−30.09 to 88.87	0.328
LA	53.09	−82.39 to 188.57	0.437

*Abbreviations: BLDI, dorsal and intermediate basolateral; CEN, central nucleus; CMN, cortical and medial nuclei; LA, lateral nucleus; CI, Confidence Interval. *p* < 0.05 significant values highlighted in bold*.

### Behavioral Results of Food Choice Task

Descriptive data for ratings and RT are included in Supplementary Figure 1. The marginal effects from the LME model for ratings showed a significant interaction of food attributes (health, taste) and image calorie type (high calorie, low calorie; *F*_(2,207)_ = 435.51, *p* < 0.001; Supplementary Table 1). No associations were seen between WHtR (*F*_(1,67)_ = 1.20, *p* = 0.27) or age (*F*_(1,67)_ = 1.55, *p* = 0.22) and rating outcomes. *Post hoc* comparisons showed participants rated high-calorie food stimuli less healthy than low-calorie food stimuli (*p* < 0.001). However, participants rated the high and low-calorie food stimuli similar for tastiness (*p* = 0.83). The marginal effects of the LME models for RT showed trend level associations with WHtR (*F*_(1,67)_ = 3.90, *p* = 0.05), and age (*F*_(1,67)_ = 3.67, *p* = 0.06), with faster RTs seen for both higher WHtR and age. Also, the marginal effect of food attribute was significant (*F*_(1,207)_ = 24.20, *p* < 0.001) with faster RT seen for the taste vs. health conditions. In terms of rating how much they would like to eat each food item, no association was found between WHtR or age (*p*-values > 0.80) and ratings, but significantly faster RT was seen with age (*F*_(1,67)_ = 4.68, *p* < 0.034) as well as a significant interaction was seen between WHtR and image calorie type for RT (*F*_(1,69)_ = 4.61, *p* < 0.035). *Post hoc* analyses showed that those at 1 SD below the mean in WHtR had significantly faster RTs for rating how much they would like to eat the low-calorie vs. high-calorie items (*p* = 0.01), whereas those with a higher WHtR did not show differences in RT between high- and low-calorie food items when rating them on how much they would like to eat each item (*p*-values > 0.15). SCSR had an average of 0.32 and ranged from 0.01 to 0.97. In terms of the decision-making portion of the task, no association was seen between SCSR and age (*β* = −0.0004, SE = 0.009, *p* = 0.96) or SCSR and WHtR (*β* = 1.46, SE = 2.37, *p* = 0.98).

### Relationships Between WHtR Identified Brain Phenotypes and SCSR

**Figure 3:**
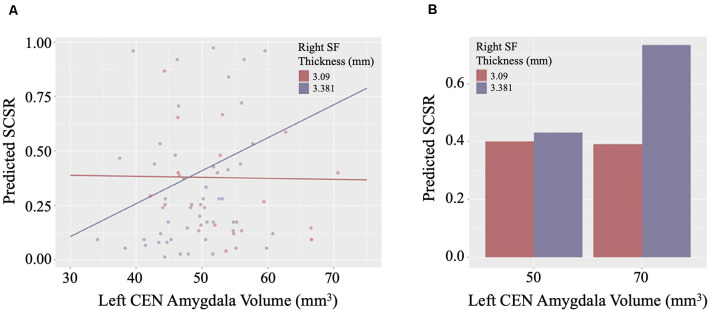
**(A)** Scatterplot of the interaction of right superior frontal (SF) cortical thickness and left central nucleus (CEN) amygdala volume on the prediction of self-control success ratio (SCSR). **(B)** Larger amygdala volume together with thinner SF thickness predicts lower SCSR. Values plotted at the first and third quartiles for both CEN volumes and SF thickness.

Linear regression models examining WHtR related brain structures of cortical thickness, CEN amygdala volumes, and/or their interactions on SCSR showed a significant left CEN amygdala-by-right superior frontal thickness interaction (Table 4, Figure 3A). A larger left CEN was only associated with poorer SCSR in conjunction with a thinner superior frontal cortex (Figure 3B). Trend level interactions were also seen for the left CEN amygdala-by-right superior frontal thickness and the left CEN-by-right inferior temporal thickness (Table 4). Last, stepwise regression including each cortical region left CEN amygdala volume, as well as age, sex, WHtR, and ICV as covariates, revealed the best predictors of SCSR included a larger left CEN amygdala volume, reduced cortical thickness of the right insula, and superior frontal thickness, as well as the significant left CEN amygdala by right superior frontal thickness (Table 5); albeit the model only accounted for ~11% of the variance in behavior on the task (*R*^2^ = 11.30, Model *p* = 0.09).

**Table 4:** Significant interactions in predicting self-control success ratio.

Interaction terms	Estimates	95% CI	*p*	*R*^2^
Left CEN-by-Left Superior Frontal thickness	0.05555	−0.00492–0.11602	0.071	0.08
Left CEN-by-Left Superior Temporal thickness	0.02172	−0.03400–0.07744	0.439	0.051
Left CEN-by-Right Insula thickness	0.02842	−0.02374–0.08058	0.28	0.059
Left CEN-by-Right Superior Frontal thickness	0.05365	0.00310–0.10421	**0.038**	0.10
Left CEN-by-Right Inferior Temporal thickness	0.04058	−0.00447–0.08564	0.077	0.103

*Abbreviations: CEN, central nucleus; CI, confidence interval. *p* < 0.05 significant values highlighted in bold*.

**Table 5:** Stepwise regression identified best predictors of self-control success ratio.

Predictors	Estimates	95% CI	*p*
Left CEN Amygdala	−0.16167	−0.31680–-0.00655	**0.041**
Right Superior Frontal Thickness	−2.14616	−4.65587–0.36356	0.092
Right Insula Thickness	−0.4481	−0.96347–0.06727	0.087
Left CEN-by-Right Superior
Frontal Thickness	0.05175	0.00337–0.10013	**0.036**

*CEN, central nucleus; CI, Confidence Interval. p < 0.05 significant values highlighted in bold*.

### Results Using BMIz

BMIz and WHtR was highly correlated (*r* = 0.71, *p* < 0.0001). Brain and behavior analyses utilizing BMIz were found to be relatively similar, yet less robust as compared to WHtR. Specifically, we found that BMIz was negatively associated with thickness in the superior frontal left PFC while controlling for age and sex (Supplementary Figures 2, 3). No association was seen between BMIz and overall amygdala volume after adjusting for the hemisphere, age, sex, and ICV. In subregion analyses, we found a BMIz-by-age interaction on the volume of the CEN, but not the CMN or LA of the amygdala, after adjusting for the hemisphere, age, sex, and ICV (Supplementary Table 4). Linear regressions found no association between SCSR and age (*β* = −0.01, SE = 0.13, *p* = 0.94), BMIz (*β* = 0.001, SE = 0.01, *p* = 0.95), or an age-by-BMIz interaction (*β* = −0.0003, SE = 0.01, *p* = 0.97). However, a significant interaction of left superior frontal PFC thickness and CEN amygdala volume was found across all participants in predicting dietary self-control, controlling for BMIz, hemisphere, age, sex, and ICV (Supplementary Table 5).

## Discussion

We studied children and adolescents of varying weight categories who each completed a dietary decision-making task and brain structural scans. We aimed to investigate brain structural correlates of obesity and dietary self-control across adolescence. We found that the WHtR was negatively associated with the cortical thickness of the bilateral superior frontal, left superior temporal, right insula, and right inferior temporal regions, and positively correlated with the volume of CEN of the amygdala. Furthermore, thinner cortical thickness of right superior frontal and larger left CEN volume predicted lower dietary self-control. These results suggested that weight status is related to the structural morphology of the PFC and central nucleus of the amygdala, which together predicted dietary self-control in children and adolescents.

We and others have observed a negative correlation between BMIz and PFC cortical thickness in youth (Laurent et al., 2020; Ronan et al., 2020). Interestingly, when WHtR was examined, we observed additional clusters exhibiting this negative relationship with PFC thickness, including left superior temporal, right insula, right inferior temporal regions. Additional clusters with WHtR (vs. BMIz) have also been noted by others in brain-obesity relationships (Ronan et al., 2020). WHtR is a surrogate marker of central obesity and is more directly associated with cardio-metabolic risk factors than BMI, thereby perhaps capturing increased variance of cortical thickness as it relates to obesity. Also, WHtR is closely associated with insulin resistance, which is related to a thinner PFC (Ross et al., 2015), and inflammation which can directly impact brain morphometry (Adelantado-Renau et al., 2019). Similarly, visceral abdominal fat, but not BMI, is related to cortical thickness, suggesting that the central allocation of fat matters in these relationships as well (Saute et al., 2018). Limited by sample size and tight correlations between BMIz and WHtR, we were not able to test the independent or joint effect of BMI and WHtR on cortical thickness and brain volumes as others have done (Hamer and Batty, 2019). The biological meaning of reduced cortical thickness is not yet fully understood, however, there is suggestive evidence showing that reduced cortical thickness may be partially associated with increases in cortical myelination (Croteau-Chonka et al., 2016). Future studies are merited to establish a link between obesity and cortical myelination by directly assessing myelination.

Additionally, we found a significant positive relationship between WHtR and volume of CEN of the amygdala, controlling for covariates such as age, sex, hemisphere, and ICV. There are mixed results in previous studies, with some showing positive relationships between obesity and amygdala volume (Perlaki et al., 2018), others showing the opposite pattern (Nouwen et al., 2017). Controversial findings could be due to differences in study populations, false-positive results due to small sample sizes, and differential contributions from subregions of the amygdala. Given the distinct structure and function of amygdala subregions, we examined relationships between obesity and the volume of four *a priori* subregions of the amygdala and found that only the volume of CEN was significantly positively related to WHtR. The CEN is anatomically and functionally connected to the hypothalamus (Swanson and Petrovich, 1998) and bed nuclei of stria terminalis (Dong et al., 2001), suggesting that its role in feeding could be *via* metabolic and/or reward regulation mechanisms. Animal work has shown that the CEN is critical for controlling feeding in the presence of an aversive cue (Petrovich et al., 2009; Prévost et al., 2012), and can modulate dopamine activity in the nucleus accumbens and medial PFC (Ahn and Phillips, 2002), suggesting that it may integrate motivation signals in cue-triggered feeding. Our study results highlight the importance of examining the subregions of the amygdala concerning obesity.

To further understand the functional significance of obesity-related brain structures reported above, we examined relationships between those brain structures and dietary self-control on an objective task. Dietary self-control is the ability to forgo tastier food items for healthier food options (Ha et al., 2016). Prior functional imaging studies in adults and youth have shown that the dorsolateral PFC was activated when healthy food choices were made; it modulates the ventromedial PFC activity when self-control was engaged (Hare et al., 2009; Lim et al., 2016). Decreased PFC activation could reduce the modulatory control of the PFC over reward regions, including the orbitofrontal cortex and striatum as well, thereby further reducing dietary self-regulation (Lowe et al., 2019). Furthermore, a meta-analysis reported a negative relationship between brain activity in the PFC cortex such as IFG and MFG during dietary self-control tasks and BMI (Han et al., 2018), suggesting that obesity may be related to impairments in dietary self-control implemented by the various parts of the PFC.

The amygdala may also play a critical role in dietary self-control. The role of the amygdala on dietary decisions is largely unexplored, although both animal and human studies suggest that the amygdala is crucial in regulating food intake (Sawa et al., 1954; Weiskrantz, 1956; Green et al., 1957; Koikegami et al., 1958; Wood, 1958; Wilkinson and Peele, 1962; Kling and Dunne, 1976). The amygdala is thought to integrate sensory, metabolic, and higher-order control signals in cue-triggered feeding behavior given its connection with regions implicated in sensory processing, homeostatic regulation, and cognitive control (Price, 2003). We speculate that the amygdala may be implicated in tracking the rewarding value of food stimuli using various input from sensory, metabolic, and top-down control signals. Prior functional imaging studies have shown that the amygdala is responsive to appetitive food cues in children (Holsen et al., 2005) and that children with obesity showed greater amygdala responses to food reward than lean healthy children (Boutelle et al., 2015). Moreover, greater amygdala volume in newborns was predictive of lower impulse control at 2 years of age (Graham et al., 2018), suggesting a link between amygdala volume and impulse control. Consistent with observations from both functional and structural brain studies, we found that a thinner right superior PFC together with a larger left CEN volume was associated with poorer dietary self-control. Our findings demonstrate that the interaction between the PFC and the amygdala is an important aspect of the regulation of food intake.

Unexpectedly, we did not observe significant relationships between dietary self-control and BMIz or WHtR. Similarly, a recent study reported no significant relationship between dietary self-control and BMI percentile in children age between 7 and 11 years old (Pearce et al., 2020). However, we did observe that participants with a WHtR that was 1 SD below the mean had significantly faster RTs for ratings of “how much they would like to eat” the high-calorie vs. low-calorie food items, whereas those with a higher WHtR did not show differences in RT between high- and low-calorie food items in this regard. These results suggest that individuals of a healthy weight may experience a greater conflict between rating “liking” of high- vs. low-calorie foods due to prioritizing the health attribute of food. We also speculate that the effect size of the association between dietary self-control and obesity may be small, thereby we would not have the power to detect significant relationships between dietary self-control and BMIz or WHtR. It is also possible that obese and lean people may make similar food choices in a fasted state. As well, dietary self-control and obesity may not be related, and changes in the brain structure of the PFC and amygdala may relate independently to these factors.

Our study has several limitations. First, the cross-sectional design limits our ability to test causal relationships between brain regions, obesity, and dietary self-control. A longitudinal study could help address these questions. Second, although WHtR is a common index of central obesity, it is not a direct quantification of visceral fat measured by MRI. Third, our study sample is relatively small, yet is composed of individuals spanning a wide age-range (8–22 years). Although we did not find significant BMI or WHtR-by-age interaction on brain structures and dietary self-control, larger studies with additional participants per age group (children, adolescents, adults) are needed to more fully examine interactions of obesity and age on brain structure and dietary self-control. There might also be other important regions relevant to dietary decisions and obesity, such as the ventral striatum, which we did not investigate here. Last, given the prominent role of the amygdala in emotion processing, it is possible that the amygdala may be important for the emotional aspects of eating behaviors, which we did not have measures to probe here. Future studies are merited to study the relationships between the amygdala morphology and emotional eating.

In conclusion, the differential development of the PFC and amygdala relate to both obesity and dietary self-control. Adolescents have the largest differential development of PFC and limbic regions. As such, this age group may be particularly susceptible to making poor dietary choices, as seen by studies that report adolescents eat more fast food and refined sugars than any other age group (Nielsen et al., 2002; Bremer and Lustig, 2012). Further longitudinal studies are merited to determine if altered PFC to amygdala neural circuitry is a cause or a consequence of dietary self-control in youth, in addition to being a risk factor for obesity.

## Data Availability Statement

The raw data supporting the conclusions of this article will be made available by the authors, upon reasonable request.

## Ethics Statement

The studies involving human participants were reviewed and approved by IRB Human Subjects Protection Office of USC and CHLA (CHLA-14-00191 and HS-16-00978). Written informed consent to participate in this study was provided by the participants’ legal guardian/next of kin.

## Author Contributions

MH, SL, and MK take responsibility for the integrity of the data in the study and the accuracy of the data analysis and concept and design. MK, SL, AA, CC, KF, and MH: drafting of the manuscript. SL and MH: statistical analysis. MK and MH: obtained funding and supervision. AA, CC, KF, and RK: administrative, technical, or material support. All authors: acquisition, analysis, or interpretation of data and critical revision of the manuscript for important intellectual content. All authors contributed to the article and approved the submitted version.

## Conflict of Interest

The authors declare that the research was conducted in the absence of any commercial or financial relationships that could be construed as a potential conflict of interest.

## Footnotes

^1^http://surfer.nmr.mgh.harvard.edu

^2^http://www.psychtoolbox.org
