# Supplementary material for: Prefrontal Cortex and Amygdala Subregion Morphology Are Associated With Obesity and Dietary Self-control in Children and Adolescents
Source: Front Hum Neurosci. 2020 Dec 3;14:563415. doi: 10.3389/fnhum.2020.563415 (PMC7744283; doi:10.3389/fnhum.2020.563415)
Supplement: Supplementary file 1 [file Data_Sheet_1.pdf]

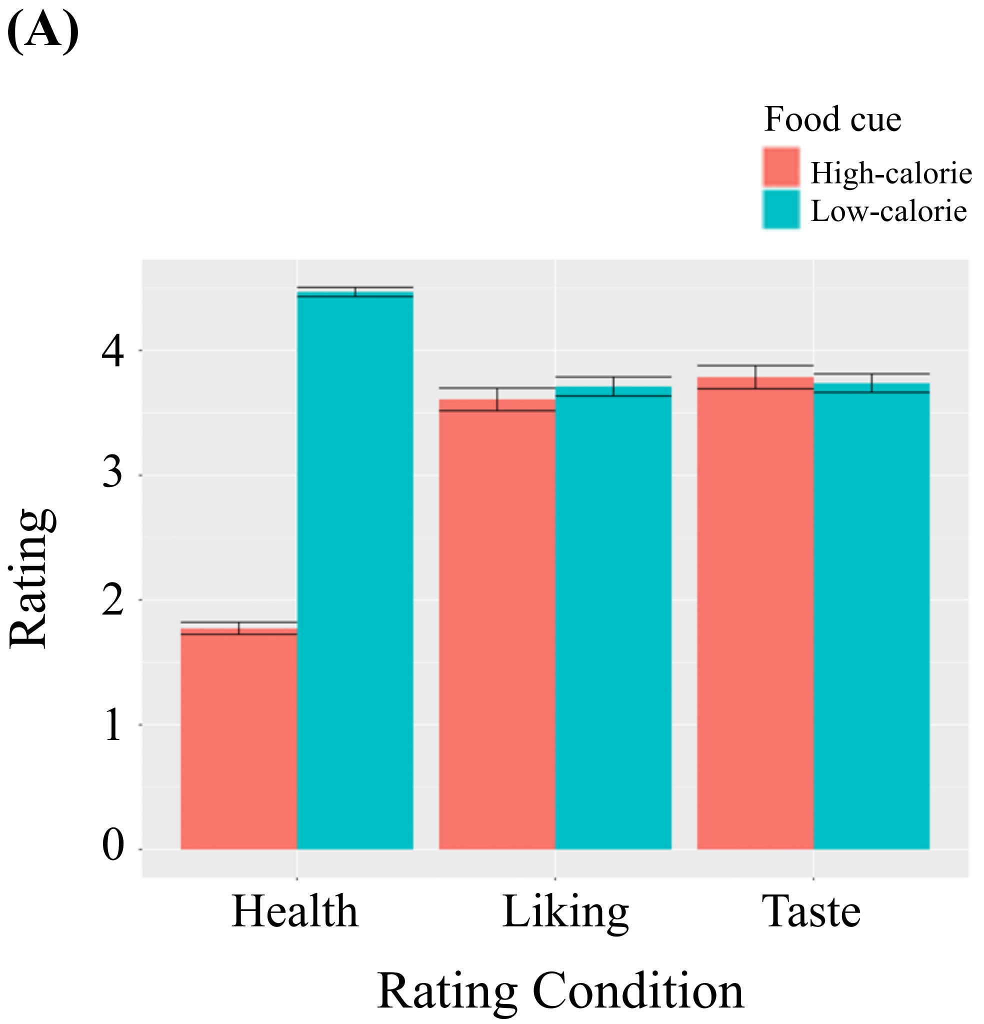

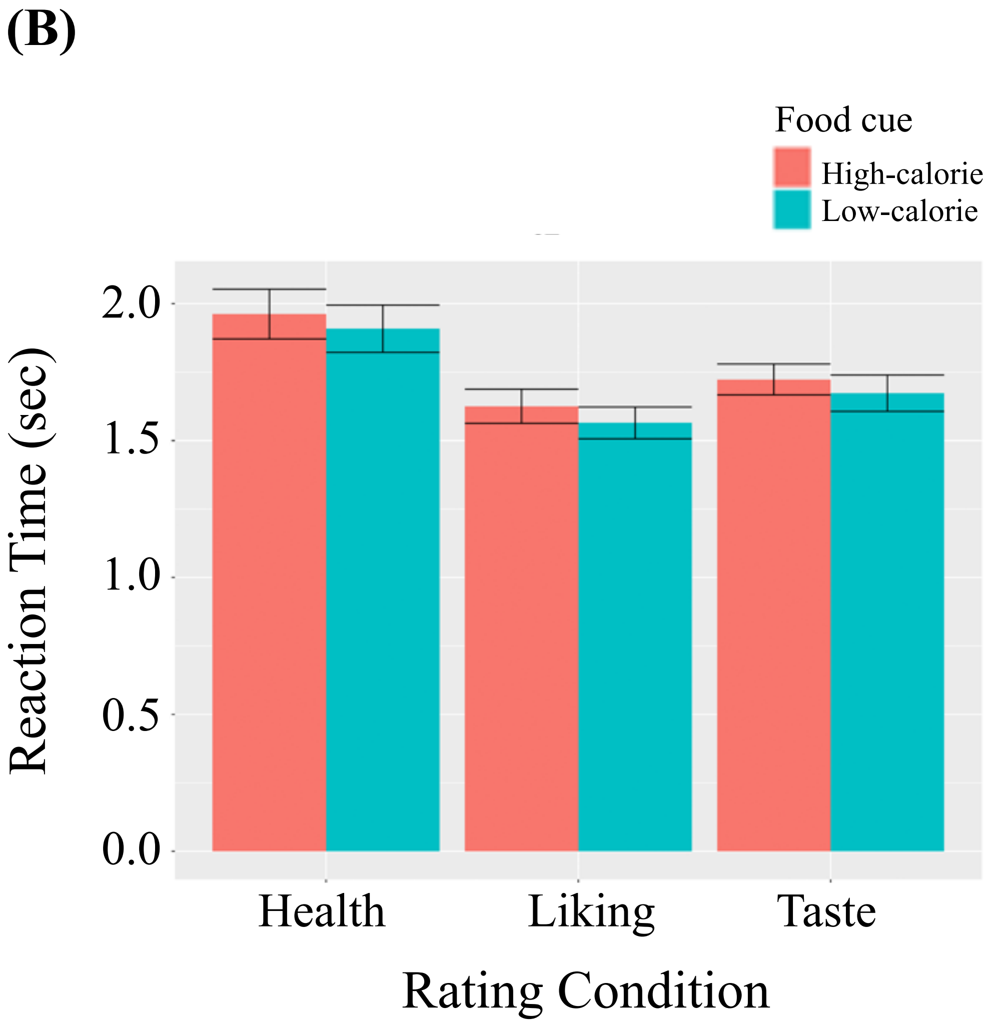

SFigure 1. Ratings and reaction times for three rating conditions (health, liking, and taste) of 60 food cue stimuli (30 high-calorie, 30 low-calorie).

Body Mass Index z-score in Relation to Cortical Thickness

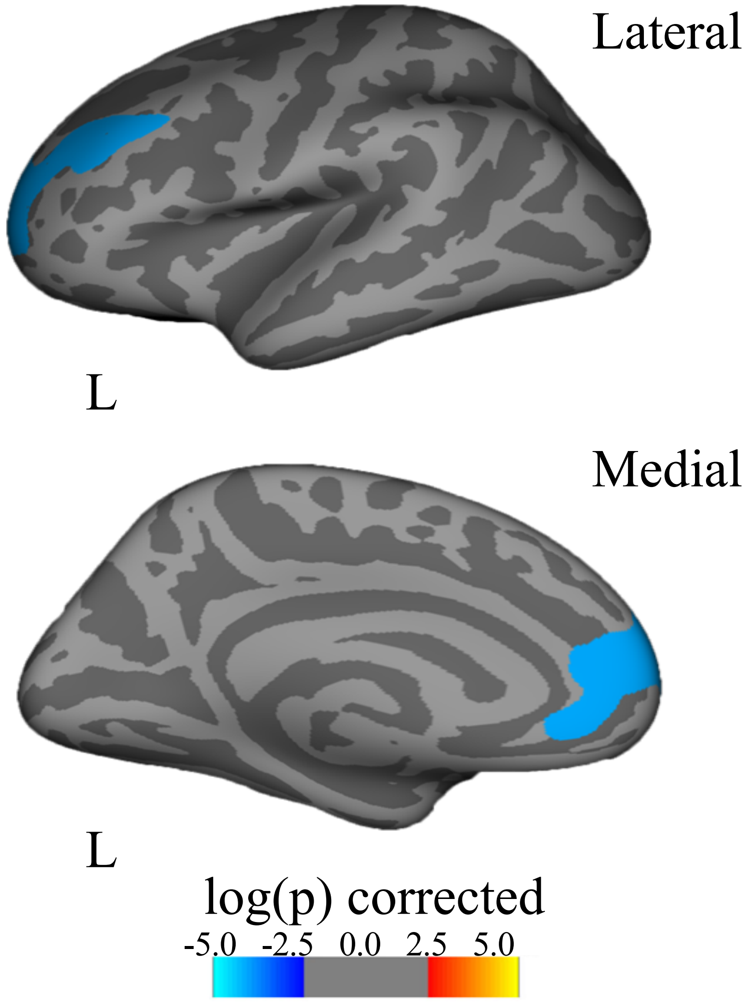

SFigure 2. Negative associations between BMIz and cortical thickness adjusting for age and sex (multiple comparisons corrected at $p < 0.05$)

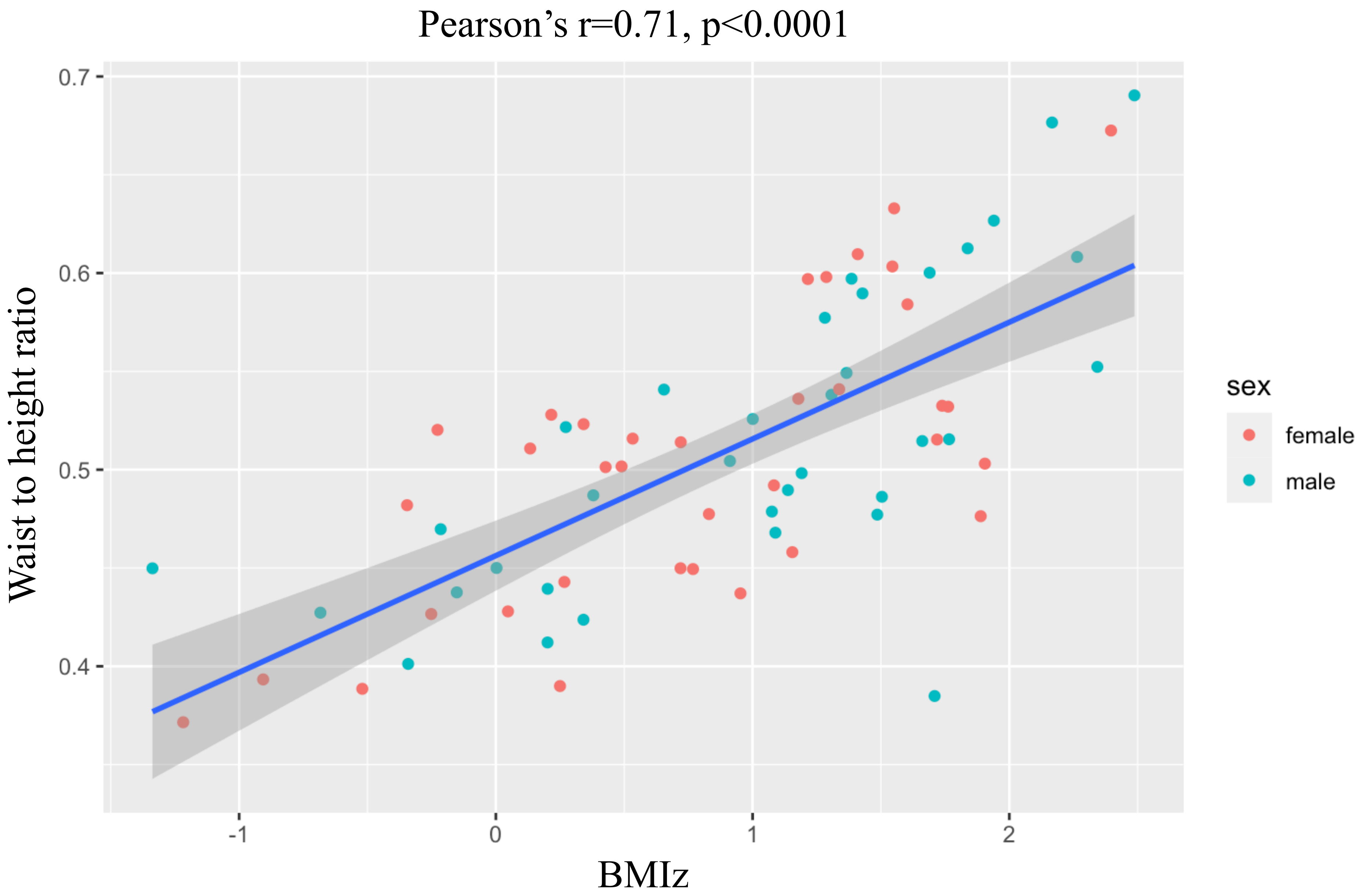

SFigure 3. Associations between BMIz and Waist-to-Height Ratio

STable 1. Ratings of Taste and Health

Marginal Effects of the LME Model of Ratings for Health & Taste

	numDF	denDF	F-value	*p*
Rating condition (health, taste)	1	207	95.229	**< 0.0001**
Food cue (high-calorie, low-calorie)	1	207	405.704	**< 0.0001**
Waist-to-height ratio	1	67	1.195	0.278
Age at study visit	1	67	1.552	0.217
Sex	1	67	0.002	0.963
Rating condition by Food cue	1	207	435.509	**< 0.0001**
Rating condition by Waist-to-height ratio	1	207	3.475	0.064
Food cue by Waist-to-height ratio	1	207	0.713	0.399
Rating condition by Food cue by Waist-to-height ratio	1	207	1.359	0.245

Marginal Effects of the LME Model of RT for Health & Taste Ratings

	numDF	denDF	F-value	*p*
Rating condition	1	207	24.252	**< 0.0001**
Food cue	1	207	1.164	0.282
Waist-to-height ratio	1	67	3.902	0.052
Age at study visit	1	67	3.627	0.061
Sex	1	67	1.157	0.286
Rating condition by Food cue	1	207	0.002	0.969
Rating condition by Waist-to-height ratio	1	207	0.004	0.949
Food cue by Waist-to-height ratio	1	207	1.619	0.205
Rating condition by Food cue by Waist-to-height ratio	1	207	0.146	0.703

STable 2. Ratings of “Like to Eat”

Marginal Effects of the LME model of Liking Ratings

	numDF	denDF	F-value	*p*
Food cue (high-calorie, low-calorie)	1	69	0.737	0.394
Waist-to-height ratio	1	67	0.046	0.831
Age at study visit	1	67	0.063	0.803
Sex	1	67	0.129	0.720
Food cue by Waist-to-height ratio	1	69	0.066	0.798

Marginal Effects of the LME model of Reaction Time for Liking Ratings

	numDF	denDF	F-value	*p*
Food cue (high-calorie, low-calorie)	1	69	2.034	0.158
Waist-to-height ratio	1	67	3.764	0.057
Age at study visit	1	67	4.684	**0.034**
Sex	1	67	0.331	0.567
Food cue by Waist-to-height ratio	1	69	4.613	**0.035**

STable 3. Negative Associations between BMI z-score and Cortical Thickness

**Cluster Location**	**Hemisphere**	**Cluster size (mm^2)**	**MNI Coordinates**		
			**X**	**Y**	**Z**
Superior Frontal Cortex	L	1369.15	-14.6	59.4	13.8

STable 4. Associations Between BMIz and Amygdala Volumes

**Amygdala Volumes (mm^3)**	**Estimates**	**95% CI**	***p***
Main Effect of BMIz			
Total Amygdala	29.68	-5.86 – 65.23	0.10
Dorsal and intermediate basolateral (BLDI)	3.28	-1.06 – 7.62	0.13
Cortical and Medial Nuclei (CMN)	0.71	-3.16 – 4.58	0.72
Lateral Nucleus (LA)	5.80	-2.88 – 14.48	0.19
BMIz-by-age Interaction			
Central Nucleus (CEN)	0.24	0.02 – 0.47	0.03

STable 5. BMIz Related Cortical Thickness and Brain Volume Effects on SCSR Controlling for BMIz

**Predictors for models**	**Estimates**	**95% CI**	***p***	**R^2**
Left Superior Frontal thickness	0.053	-0.417 – 0.523	0.823	0.022
Right Central Nucleus Volume	0.005	-0.007 – 0.018	0.404	0.036
Left Central Nucleus Volume	-0.001	-0.016 – 0.013	0.863	0.026
Right Central Nucleus Volume-by-Left Superior Frontal thickness	0.036	-0.015 – 0.086	0.161	0.058
Left Central Nucleus Volume-by-Left Superior Frontal thickness	0.050	0.007 – 0.094	**0.024**	0.114
